# Spatial Intracranial Pressure Fields Driven by Blast Overpressure in Rats

**DOI:** 10.1007/s10439-024-03544-7

**Published:** 2024-06-08

**Authors:** Carly Norris, Susan F. Murphy, Caiti-Erin Talty, Pamela J. VandeVord

**Affiliations:** 1 School of Biomedical Engineering and Sciences, Virginia Tech, Blacksburg, VA USA; 2 Department of Biomedical Engineering and Mechanics, Virginia Tech, Blacksburg, VA USA; 3 Veterans Affairs Medical Center, Salem, VA USA; 4 Graduate Program in Translational Biology, Medicine and Health, Virginia Tech, Blacksburg, VA USA

**Keywords:** Intracranial pressure, Blast-induced traumatic brain injury, Injury characterization, In vivo, Biomechanics

## Abstract

Free-field blast exposure imparts a complex, dynamic response within brain tissue that can trigger a cascade of lasting neurological deficits. Full body mechanical and physiological factors are known to influence the body’s adaptation to this seemingly instantaneous insult, making it difficult to accurately pinpoint the brain injury mechanisms. This study examined the intracranial pressure (ICP) profile characteristics in a rat model as a function of blast overpressure magnitude and brain location. Metrics such as peak rate of change of pressure, peak pressure, rise time, and ICP frequency response were found to vary spatially throughout the brain, independent of blast magnitude, emphasizing unique spatial pressure fields as a primary biomechanical component to blast injury. This work discusses the ICP characteristics and considerations for finite element models, in vitro models, and translational in vivo models to improve understanding of biomechanics during primary blast exposure.

**Supplementary Information:**

The online version contains supplementary material available at 10.1007/s10439-024-03544-7.

## Introduction

Defining the injury mechanics leading to primary blast-induced traumatic brain injury (bTBI) is not straightforward considering the entire body is exposed to the blast, leading to diffuse pathologies and a full systemic response [1]. As such, a number of theories have been proposed to explain large-scale bTBI mechanics: direct intracranial transmission, skull flexure, skull orifices, and thoracic surge [2, 3]. Although it is possible that a combination of these mechanisms contributes to the injury, researchers have failed to reach a consensus. bTBI mechanisms would best be defined through direct correlation with the stresses and strains imparted on the brain tissue during blast exposure, which motivated the development of human computational models [3–7]. Nevertheless, collection and validation of such measurements in humans remains infeasible. Therefore, animal models are employed to measure biomechanical and pathophysiological effects of blast exposure and these responses are then used to infer outcomes in the human brain [8–11].

Real-time monitoring of the change in intracranial pressure (ICP) during blast exposure was adopted to best interpret the brain mechanics. Experimental changes in ICP during blast have been measured in rats [12–21] and pigs [22–24]. Experimental ICP may be used to validate computational models and predict the associated brain stresses and strains in these animal models [19, 24–26]. However, most experimental analysis focuses on changes in the peak ICP, as opposed to the full ICP profile. For instance, researchers have shown that ICP magnitudes are highly variable and are sensitive to subject orientation relative to the wave (head-on vs. side-on), blast overpressure magnitude, animal age, and animal weight [3, 16, 18, 27]. Additionally, ICP measured in bTBI rat models typically lacks the spatial sensitivity necessary to control for region-specific ICP responses. Therefore, there is a significant need to improve parameterization of ICP profiles and methods to enhance spatial assessments to ultimately improve validation of increasingly complex computational models and better understand bTBI mechanics.

The purpose of this study was to characterize the full ICP profile and examine the sensitivity of these profile characteristics to changes in blast magnitude and brain location in a rat model. Characterization of the spatial biomechanical response as a function of overpressure magnitude will (1) provide relevant information as a way for in vivo models to standardize parameterization of the ICP response, (2) improve validation of finite element models, (3) inform in vitro bTBI models of the anticipated ICP profiles within the brain environment, (4) influence understanding of regional changes in pathophysiology, and (5) provide further insight into the driving bTBI injury mechanisms.

## Materials and Methods

### Animal Model

All procedures were performed following approval from the Virginia Tech Institutional Animal Care and Use Committee. Male Sprague–Dawley rats (*n* = 9; Envigo, Dublin, VA, USA) were acclimated for at least three days and followed a 12-h light-dark cycle with food and water administered ad libitum. Subjects were 10 weeks old and weighed an average of 299 ± 16 g when tested. On the day of testing, the subjects were transported from the animal facility to the Virginia Tech Center for Injury Biomechanics where the surgical procedure, blast exposure, and subsequent euthanasia occurred.

### Surgical Procedure

**Fig. 1:**
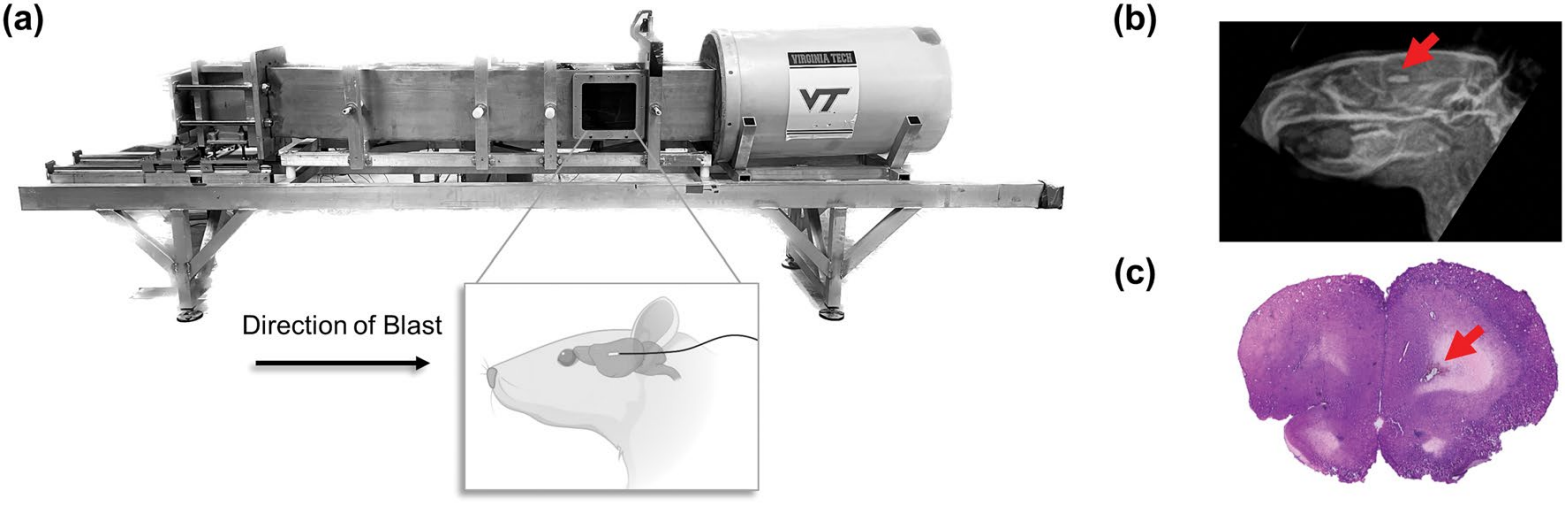
Overview of experimental procedures. **a** Sensors were surgically inserted through the occiput where sensor location was varied between subjects. Subjects were then placed in a taut mesh sling inside the 1 ft x 1 ft test section of the ABS and exposed head-on to 12 blast waves where the overpressure magnitude was varied. **b** Following the blast procedures, location of sensor placement was confirmed using biplanar X-rays and **c** H&E staining, as indicated by the red arrows

To collect real-time changes in the rat ICP as a measure of the biomechanical response during blast, a single SPR-524 Millar Mikro-Tip pressure gauge (ADInstruments, Colorado Springs, CO, USA) was surgically inserted through the occiput to minimize disruption of the wave profile or skull dynamics during the head-on blast (Fig. 1a). Sensor placement within the brain was varied by angle and depth of insertion. The SPR-524 was particularly beneficial due to its small size (tip diameter = 1.2 mm) and sensitivity (0.188 mV/kPa). Surgical methods were adapted from Dal Cengio Leonardi et al. [27] where subjects were briefly anesthetized with 4% isoflurane prior to administration of ketamine/xylazine (80/10 mg/kg IP). The animals were placed on a heating pad set to 37 °C throughout surgical procedures. Once stabilized in a stereotaxic frame, a vertical incision was made along the scalp on the back of the head to expose the occiput and surrounding musculature. Hydrogen peroxide was used to clean the skull, followed by acetone to degrease the bone surface. Surrounding musculature was then coated with Vetbond tissue adhesive (3 M, Saint Paul, MN, USA) to control bleeding. A stereotaxic high-speed drill was used to drill a 1.2 mm diameter hole, taking great care not to damage the vasculature at the back of the brain, approximately 3 mm to the right of the mid-line and 3.5 mm from the top of the skull. The sensor was then inserted and held in place while a viscous acrylate resin InstaCure + (Bob Smith Industries, Atascadero, CA, USA) was used to seal any gaps between the sensor cable and the surrounding bone at the point where the sensor exited the occiput. After sealing with the viscous acrylate resin, a wait period of approximately 1.5–2.5 h was implemented to allow for CSF production and re-pressurization within the intracranial cavity [28, 29]. The scalp was then sutured shut and reinforced with Vetbond tissue adhesive. To prevent snagging and subsequent removal of the sensor during blast exposure, the sensor was sutured to the skin on the upper neck and the torso. Quarter doses of ketamine/xylazine were administered every 30 minutes for a total of approximately 3-4 hours until all surgical and blast testing procedures were completed.

### Blast Testing

Blast procedures were conducted using an advanced blast simulator (ABS) with a 1 ft x 1 ft test section, previously characterized in Cho et al. [30] and subsequent studies investigating primary blast injuries [31–36]. The blast wave was driven by compressed helium and the magnitude was varied based on the thickness of the acetate membrane separating the driver compartment and the transition section. The window of the test section was removed and anesthetized subjects were placed in the prone position in a taut mesh sling oriented anterior to the blast wave (Fig. 1a). Once the acetate membrane ruptured, an air shockwave was channeled along the ABS, over the test section, and was absorbed by an end wave eliminator to keep the wave from reflecting onto the specimen of interest. Piezoelectric sensors (PCB Piezotronics Inc., Depew, NY, USA) in the wall of the ABS measured the static overpressure (SOP) adjacent to the test subject and the SPR-524 sensor measured the associated rat ICP.

The subjects were then exposed to three blasts at four target SOP magnitudes: 28–55 kPa, 55–96 kPa, 96–131 kPa, and 131–172 kPa. The lowest SOP magnitude was conducted three times in a row before proceeding to the next target SOP magnitude. Thus, each subject was exposed to a total of 12 blasts. Pressures ranging from 28 to 55 kPa were selected as the lower bound, which is clinically relevant to low-level blast overpressures experienced during breacher training [37]. Range-specific pathophysiology, previously reported in VandeVord et al. [38], motivated the comparison of ICP responses at SOP magnitudes between 55–96 kPa and 96–131 kPa. Lastly, the upper limit between 131 and 172 kPa was selected based on studies reporting increased mortality rates in rat models above this threshold [39]. Euthanasia immediately followed the final blast exposure via overdose with ketamine/xylazine (80/10 mg/kg IP).

### Sensor Placement Confirmation

Biplanar X-rays (axial and sagittal views) were collected, and Hematoxylin and Eosin (H&E) staining was performed following all blast procedures to confirm ICP sensor placement (Fig. 1b–c). X-rays were collected using a DynaRad HF-110A portable X-ray unit (70 kVp, 15 mA, 0.2 s) with a Mars1417V flat panel detector (iRay Technology Ltd., Shanghai, CN). Immediately following post-mortem X-ray imaging, the brain was extracted and post-fixed in 4% paraformaldehyde solution for 48 hours. The brains were then cryoprotected in a 30% sucrose solution for 72 h, embedded in Tissue-Tek (Sakura Finetek, Torrance, CA, USA) optimal cutting temperature (OCT) compound, and frozen at − 80 °C. The frozen brain tissue was cryosectioned starting at the cerebellum and moving in the anterior direction. Cryosectioning continued until the sensor path was no longer visible in each section. Sectioned tissues were stored in a well-plate at 4 °C containing 1X phosphate buffered saline with 0.05% sodium azide solution until staining procedures began. For each animal, three tissue sections from the most anterior regions containing visible evidence of tissue displacement from the sensor were mounted onto a single gelatin-coated slide per animal. Staining procedures were then performed using a H&E staining kit (Abcam, Cambridge, UK; ab245880) and images were collected on a PathScan Enabler IV digital pathology slide scanner (Meyer Instruments, Inc., Houston, TX, USA). Sensor coordinates were then charted according to the lateral midline, depth from superior to inferior region, and Bregma location as defined by the Paxinos and Watson Sprague–Dawley rat brain atlas in stereotaxic coordinates [40]. From the X-rays, sensor insertion depth and distance from the lateral midline were measured based on a known reference scale using OPAL-RAD software (Viztek, LLC, Garner, NC, USA). X-ray and H&E coordinate agreement were then assessed and charted for each subject according to Table 1.

**Table 1:** ICP sensor locations and approximated coordinates of all nine subjects based on post-mortem X-ray and H&E assessment

Subject	Brain region	Lateral midline	Depth	Bregma
1	Superior Colliculus	− 0.6 mm	− 4.0 mm	− 6.12 mm
2	Ventricle	0.0 mm	− 4.0 mm	− 4.68 mm
3	Ventricle	2.8 mm	− 3.8 mm	− 1.56 mm
4	Caudate Putamen	1.6 mm	− 4.0 mm	2.76 mm
5	Hypothalamus	− 0.8 mm	− 9.6 mm	− 3.24 mm
6	Hypothalamus	0.2 mm	− 8.8 mm	− 3.36 mm
7	Hypothalamus	0.4 mm	− 7.5 mm	− 3.60 mm
8	Thalamus	3.2 mm	− 6.2 mm	− 3.00 mm
9	Thalamus	0.4 mm	− 6.8 mm	− 2.64 mm

### Data Processing

Data was acquired at 800 kHz using a TMX Multi-Channel High Speed Data Acquisition Recorder (AstroNova Inc., West Warwick, RI, USA). A twenty percent pre-trigger was set to detect a change in 14 kPa from the wall sensors and 30 ms of data were collected for each blast. The resulting pressure profiles were exported and all data were processed in MATLAB R2022a (Mathworks, Natick, MA, USA). Offsets from baseline were corrected, however, filtering was not performed so as not to corrupt any frequency responses seen in the tissue. Wave characteristics such as the peak pressure, rise time, peak rate of change of pressure (dP/dt), positive phase duration, and positive phase impulse were measured in the SOP traces as well as the corresponding ICP profiles. A distinct frequency response observed just following the peak ICP was quantified as the average frequency between the first two oscillation periods, which was calculated manually rather than using a Fast Fourier Transform. This frequency response was only calculated in the ICP as it was not present in the SOP profiles. For data visualization, all profiles were aligned to twenty percent of the maximum value.

### Statistical Analysis

Statistical analysis was conducted using GraphPad Prism 9.5.1 (GraphPad Software, San Diego, CA, USA) where *p* < 0.05 was significant. To analyze how each ICP parameter changes as a function of increasing SOP magnitude, ICP peak pressures, rise times, peak dP/dt, positive durations, positive impulses, and ICP frequencies were averaged for each subject at each target magnitude. A repeated measures one-way ANOVA with the Geisser-Greenhouse correction was then conducted followed by Tukey’s multiple comparisons test with individual variances where p-values were multiplicity adjusted.

To determine whether ICP parameters differ between brain region, ICP peak pressures, rise times, peak dP/dt, positive durations, and positive impulses were normalized by the corresponding SOP wave parameter to remove any SOP magnitude-specific effects. ICP frequencies were not normalized. Parameters were averaged for each subject at each target magnitude and a repeated measures two-way ANOVA with the Geisser-Greenhouse correction was conducted followed by Tukey’s multiple comparisons test with individual variances where p-values were multiplicity adjusted.

## Results

### Qualitative Assessment

**Fig. 2:**
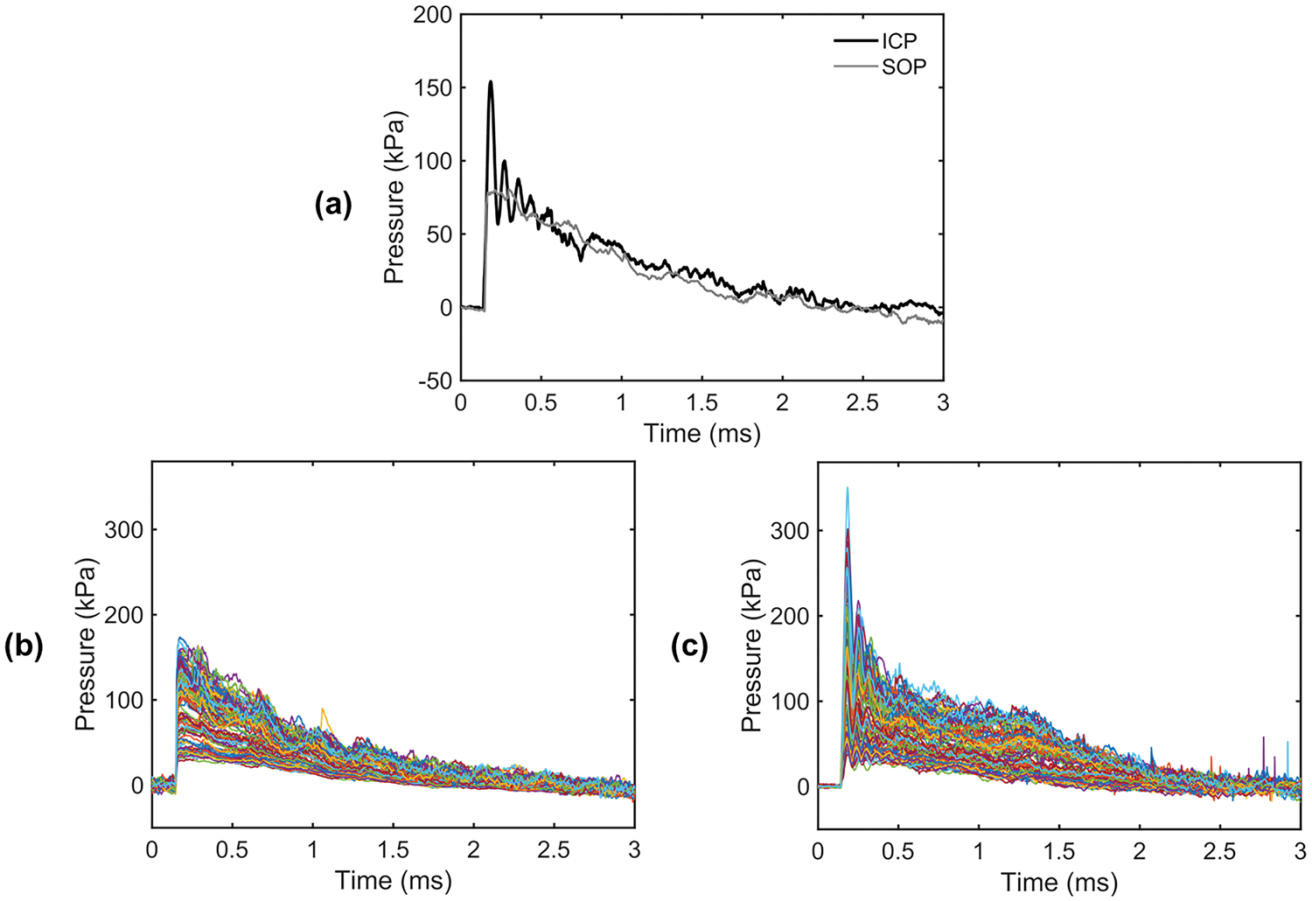
SOP and ICP profiles. **a** Representative ICP response compared to the associated SOP where an initial frequency response and peak pressure amplification in the ICP is evident. **b** Overlaid SOP profiles and **c** ICP profiles from all animals. The peak ICP was greater than the corresponding peak SOP and a distinct frequency response following the peak ICP was visible in all cases

Characteristics such as the peak pressure, rise time, peak dP/dt, positive duration, and positive impulse of the SOP and associated ICP profiles were analyzed to determine the relationships as a function of overpressure magnitude and sensor location within the brain. Representative SOP and associated ICP responses are shown in Fig. 2. The SOP curves followed a Friedlander waveform, representing a free-field explosion, which is critical for interpretation of primary bTBI biomechanics. The shape of the ICP characteristic curves exhibited a correlation with its associated SOP wave profile (Fig. 2a). However, the ICP traces contained a distinct frequency response within the first 0.2 ms of the blast, which was not present in the SOP traces.

Preliminary tests were conducted to confirm that this frequency response was not due to sensor ringing or flexing within the brain (data not shown). Sensor “ringing” refers to the vibration of the sensor wire inside the brain as a response to the blast, causing a frequency response. Sensor “flexing” refers to the deformation of the sensor tip, which could have occurred at its natural frequency during the blast. A rigid steel rod was attached to a SPR-524 Millar gauge and the reinforced sensor was inserted 10 mm through the occiput such that a portion of the rod protruded from the insertion site. The rigid attachment to the skull created a cantilever to prevent major ringing from occurring inside the brain. The frequency response remained present even with reinforcement, concluding that motion of the sensor during blast is not largely contributing to the driving intracranial pressure responses. Thus, the relationship of ICP frequency was analyzed as a function of overpressure magnitude and sensor location.

**Fig. 3:**
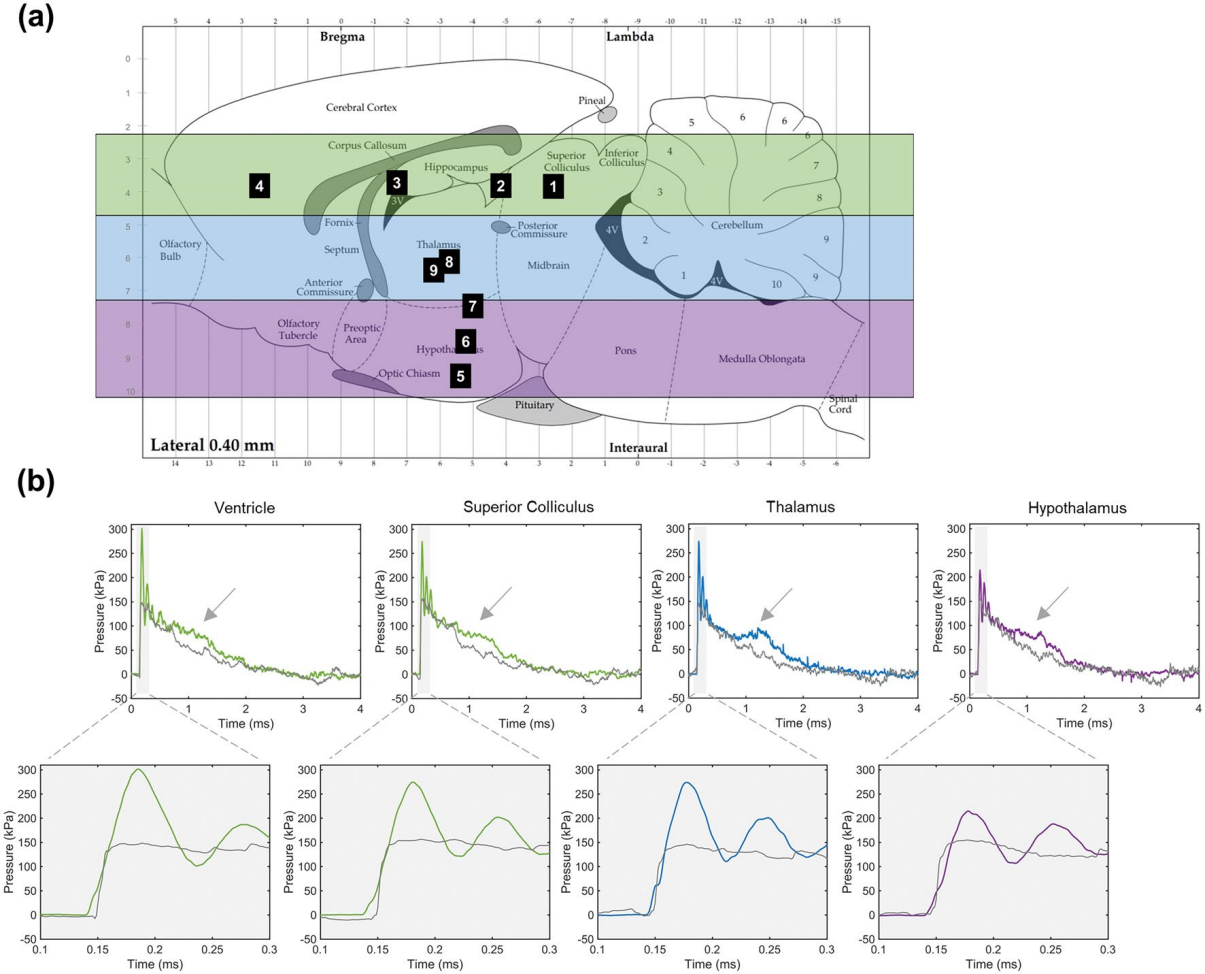
Representative pressure fields by sensor location. **a** Sensor location of each subject mapped onto the rat brain atlas and stratified by color into superior (green), midbrain (blue), and inferior (purple) brain regions. **b** Pressure fields displaying the ICP (colored according to stratified region) and corresponding SOP (gray) within the ventricle, superior colliculus, thalamus, and hypothalamus regions. The shaded sections zoom in on the first 0.15 ms and the gray arrow indicates a plateau region in the ICP

ICP profiles displayed region-specific differences (Fig. 3). The sensor numbers in Fig. 3a correspond to the subject numbers and sensor coordinates in Table 1. Qualitative trends were identified by grouping the sensors located in the superior ventricle, superior tissue, midbrain thalamus, and inferior hypothalamus regions. Visually, the ICP profiles in superior regions (green) had greater peak pressures compared to the hypothalamus region (purple) and the ICP response in the thalamus region (blue) exhibited a more pronounced plateau (Fig. 3b) compared to the other regions. Further, the plateau feature was most prominent at greater shock magnitudes. Quantitative statistical comparisons were conducted to verify such associations.

### Quantitative Analysis

#### Peak Pressure

**Fig. 4:**
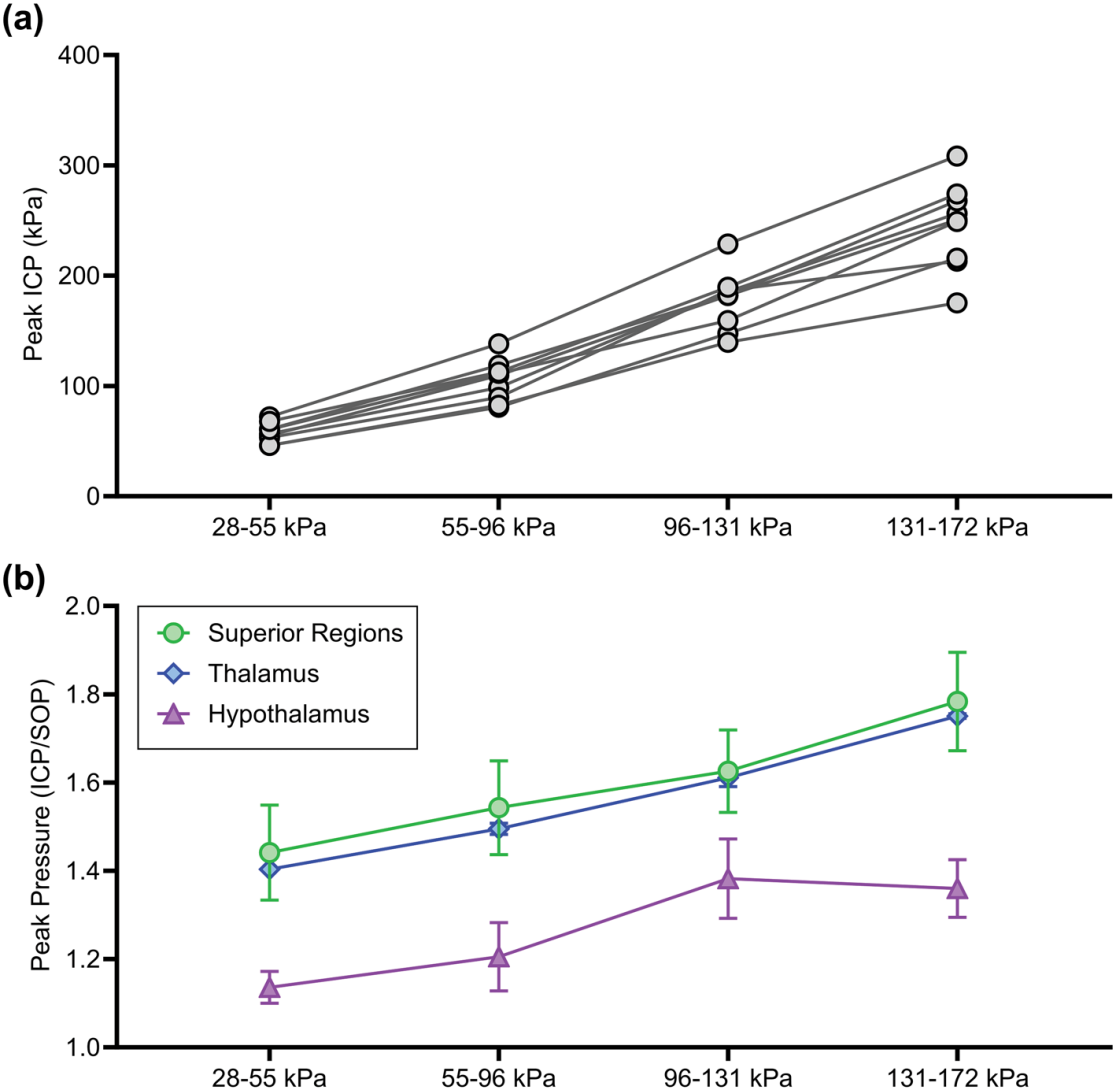
Peak ICP as a function of **a** SOP magnitude and **b** sensor location (normalized by peak SOP). Peak ICP increased with SOP magnitude for each subject (*p* < 0.0001). Average peak ICP was lower in the inferior hypothalamus region compared to the superior and midbrain thalamus regions (*p* = 0.08)

Peak ICP was greater than the corresponding peak SOP in all cases. The peak ICP increased with increasing peak SOP (Fig. 4a) where disparities became more significant at higher pressures. Further analysis showed that the ratio of peak ICP to peak SOP significantly increased with increasing SOP magnitude (Supplementary Fig. 1), which supports theories that an elastic system may be driving the ICP response. However, when analysis was performed on a per-subject basis, the relationship between peak ICP and increasing peak SOP was found to be linear (Table 2) at overpressures between 28 and 172 kPa. When analyzed independently from overpressure magnitude, peak ICP was found to be lower in the hypothalamus region compared to the thalamus and superior regions (*p* = 0.08), although not significant (Fig. 4b). At the subject-level, the factor of ICP increase was directly related to the depth of the ICP sensor relative to the top of the skull, with the exception of subject 2 (Table 2).

**Table 2:** Subject-specific linear relationships between peak ICP (Y) and peak SOP (X) ordered by depth from the top of the skull

Depth (mm)	Subject	Brain region	Linear fit equation	*R*^2^
− 3.8	*3*	Ventricle	Y = 2.14X − 3.06	0.9920
− 4.0	*1*	Superior Colliculus	Y = 1.86X − 4.19	0.9856
− 4.0	*2*	Ventricle	Y = 1.66X − 3.43	0.9966
− 4.0	*4*	Caudate Putamen	Y = 1.98X − 4.15	0.9710
− 6.2	*8*	Thalamus	Y = 1.86X − 2.95	0.9743
− 6.8	*9*	Thalamus	Y = 1.80X − 2.70	0.9714
− 7.5	*7*	Hypothalamus	Y = 1.69X − 3.36	0.9825
− 8.8	*6*	Hypothalamus	Y = 1.53X − 2.56	0.9892
− 9.6	*5*	Hypothalamus	Y = 1.30X − 1.57	0.9470

#### Rise Time

**Fig. 5:**
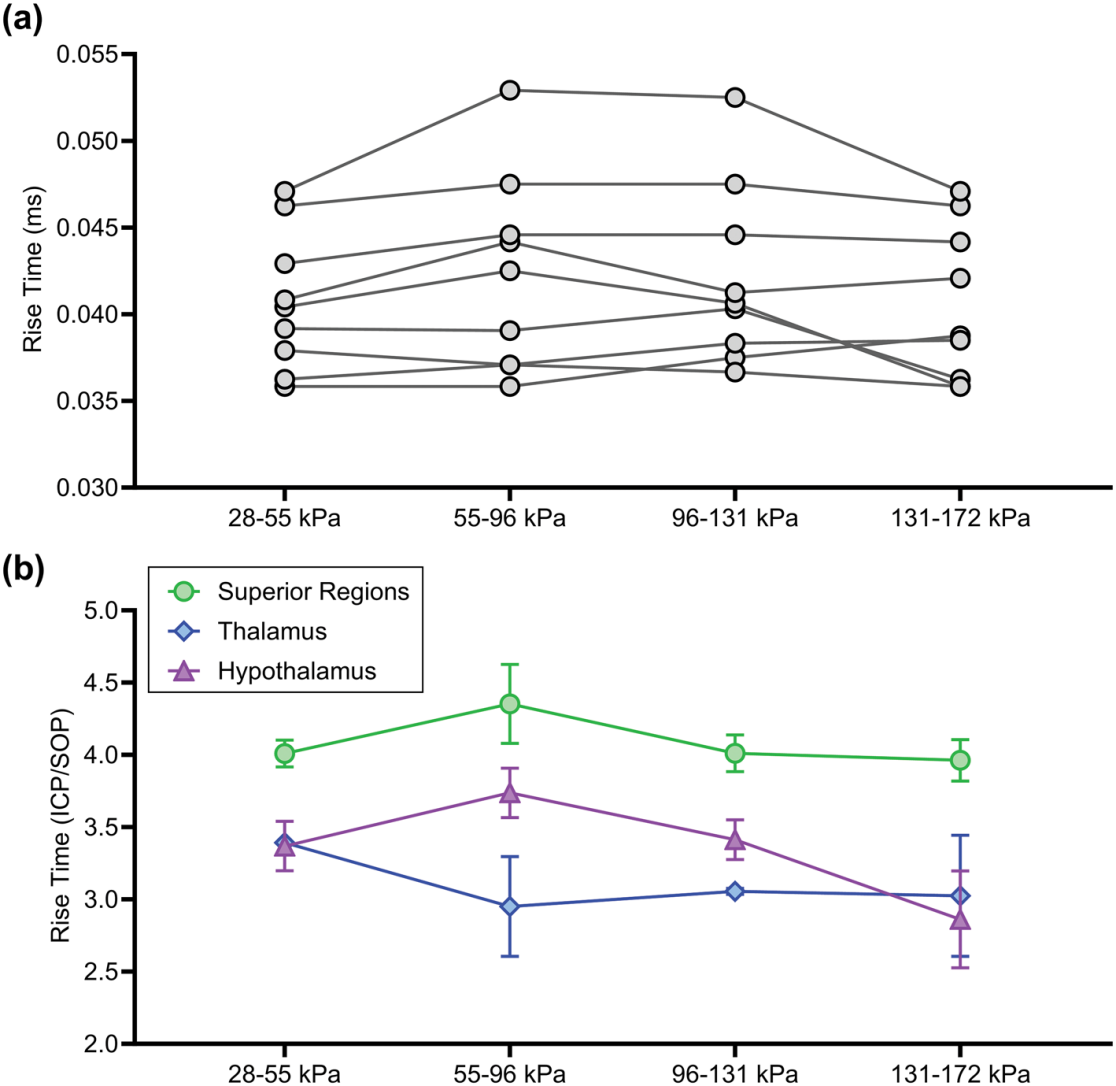
ICP rise time as a function of **a** SOP magnitude and **b** sensor location (normalized by the respective SOP rise time). ICP rise time did not significantly change with SOP magnitude and was significantly higher in the superior regions (green) compared to the thalamus and hypothalamus regions (*p* < 0.01)

The average rise time of the SOP was 12 µs and was quantified as the time it took for the pressure to increase from the baseline or ambient pressure to the peak pressure. The ICP rise times did not significantly change with increasing SOP magnitude (Fig. 5a), consistent with the nature of a shock front. However, many studies are limited by the pressure sensor sampling rate where the ‘true’ air shock rise time is in fact less than 1 µs [41, 42]. The importance of reporting it here was to demonstrate consistency in the measured SOP rise time. Alternatively, the average duration from baseline to peak ICP was 42 µs where we hypothesize that the measurement of the ICP rise time is driven by the speed of skull motion upon contact with the shock front. The ICP rise time was found to be dependent on brain region (*p* < 0.01). More specifically, superior regions (ventricle and tissue) had significantly greater ICP rise times compared to the thalamus and hypothalamus regions (Fig. 5b).

#### Peak Rate of Change of Pressure

**Fig. 6:**
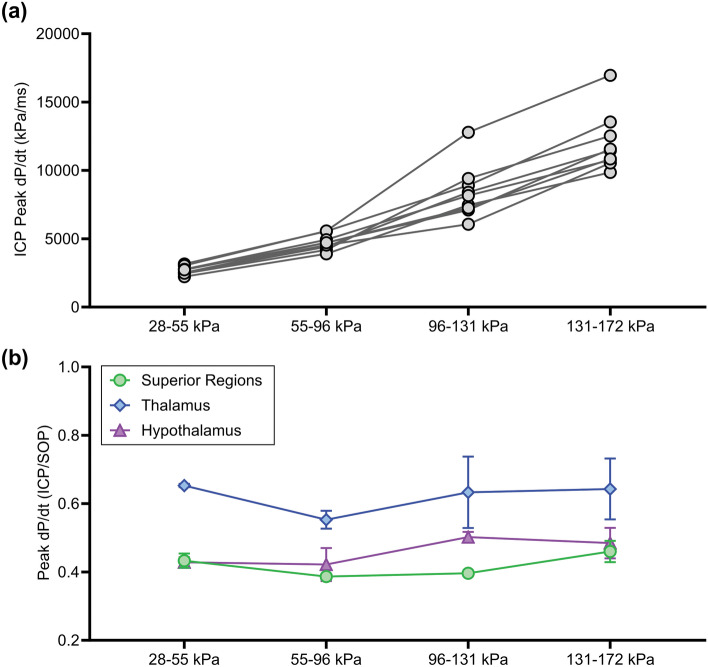
ICP peak dP/dt as a function of **a** SOP magnitude and **b** sensor location (normalized by the respective SOP peak dP/dt). ICP peak dP/dt increased with SOP magnitude (*p* < 0.0001) and was significantly higher in the midbrain thalamus region (blue) compared to the superior and inferior regions (*p* < 0.01)

The rate dependence was analyzed as the peak rate of change of pressure with respect to time (dP/dt). This sensitive measure considers the rate at which the tissue is stressed. ICP peak dP/dt significantly increased with increasing SOP magnitude (*p* < 0.0001) (Fig. 6a). ICP peak dP/dt magnitudes were approximately half that of the SOP peak dP/dt, which may be due to differences in acoustic impedance of the various regions but could also be explained by the skull flexure having different effects on the waveform at different locations. The normalized ICP peak dP/dt was found to be region-specific (*p* < 0.01) where the thalamus region exhibited a significantly greater rate of change of pressure than the surrounding regions (Fig. 6b).

#### Positive Duration and Impulse

**Fig. 7:**
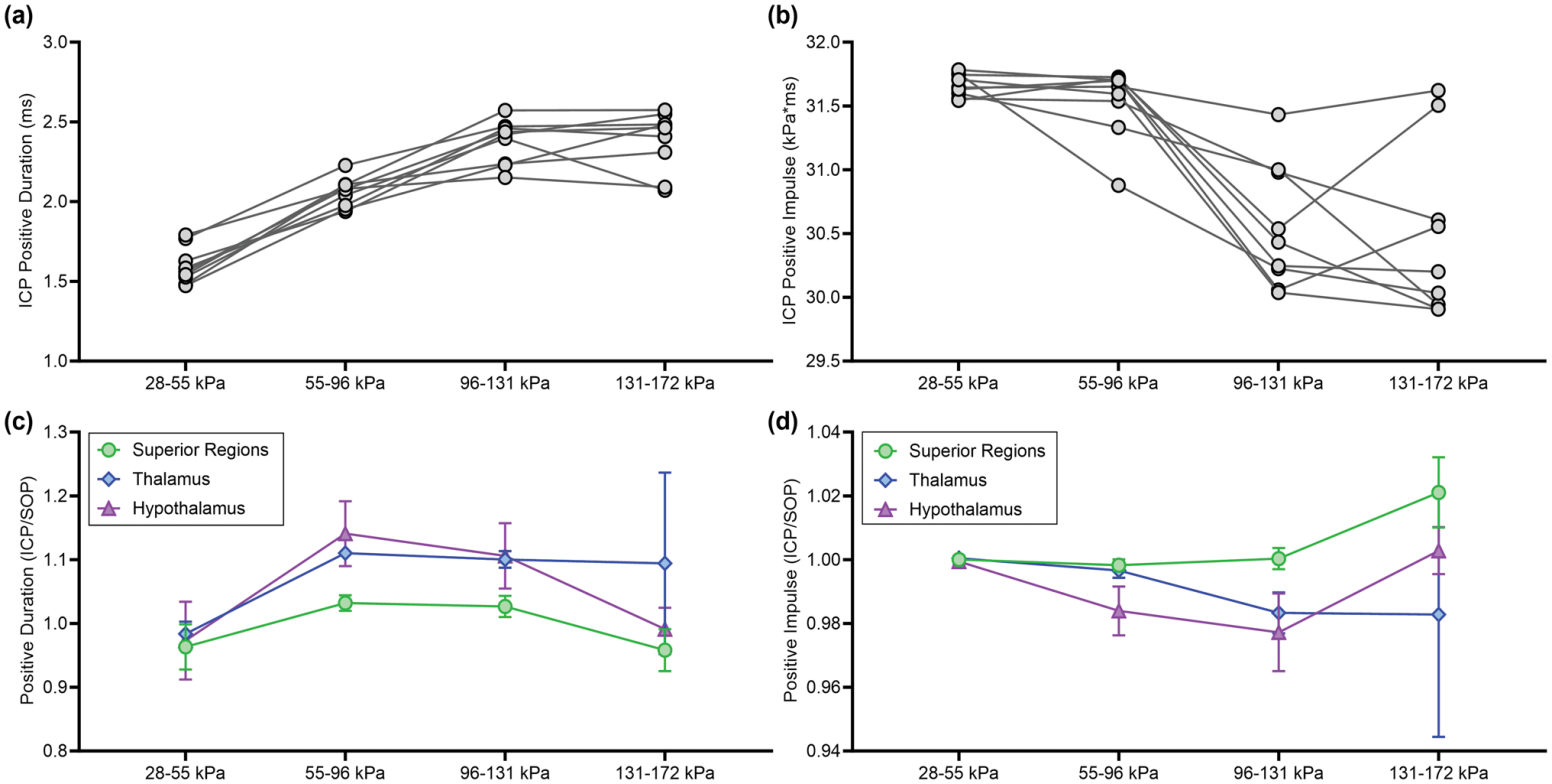
**a** ICP positive duration and **b** ICP positive impulse as a function of SOP magnitude. ICP positive duration significantly increased and ICP positive impulse significantly decreased with increasing SOP magnitude (*p* < 0.0001). Statistical analysis of **c** ICP positive duration and **d** ICP positive impulse as a function of sensor location (normalized by the respective SOP duration or impulse). Sensor location did not significantly influence metrics for ICP positive duration (*p* = 0.3) or impulse (*p* = 0.2)

The SOP magnitude had a significant effect on the ICP positive phase duration and impulse (*p* < 0.0001). ICP positive duration significantly increased as a function of peak static overpressures between 28 and 131 kPa, but there was no difference in positive durations between 96 and 172 kPa (Fig. 7a). Conversely, the ICP positive impulse significantly decreased between peak static overpressures of 28–96 kPa and 96–172 kPa (Fig. 7b). Trends in the ICP response were consistent with trends in the SOP duration and impulse. Once normalized by the SOP positive duration and impulse, these ICP measures were not sensitive to changes in sensor location (Fig. 7c, d). Together these results show that ICP positive durations and impulses were not as sensitive to changes in blast magnitude or sensor location compared to other metrics.

#### ICP Frequency

**Fig. 8:**
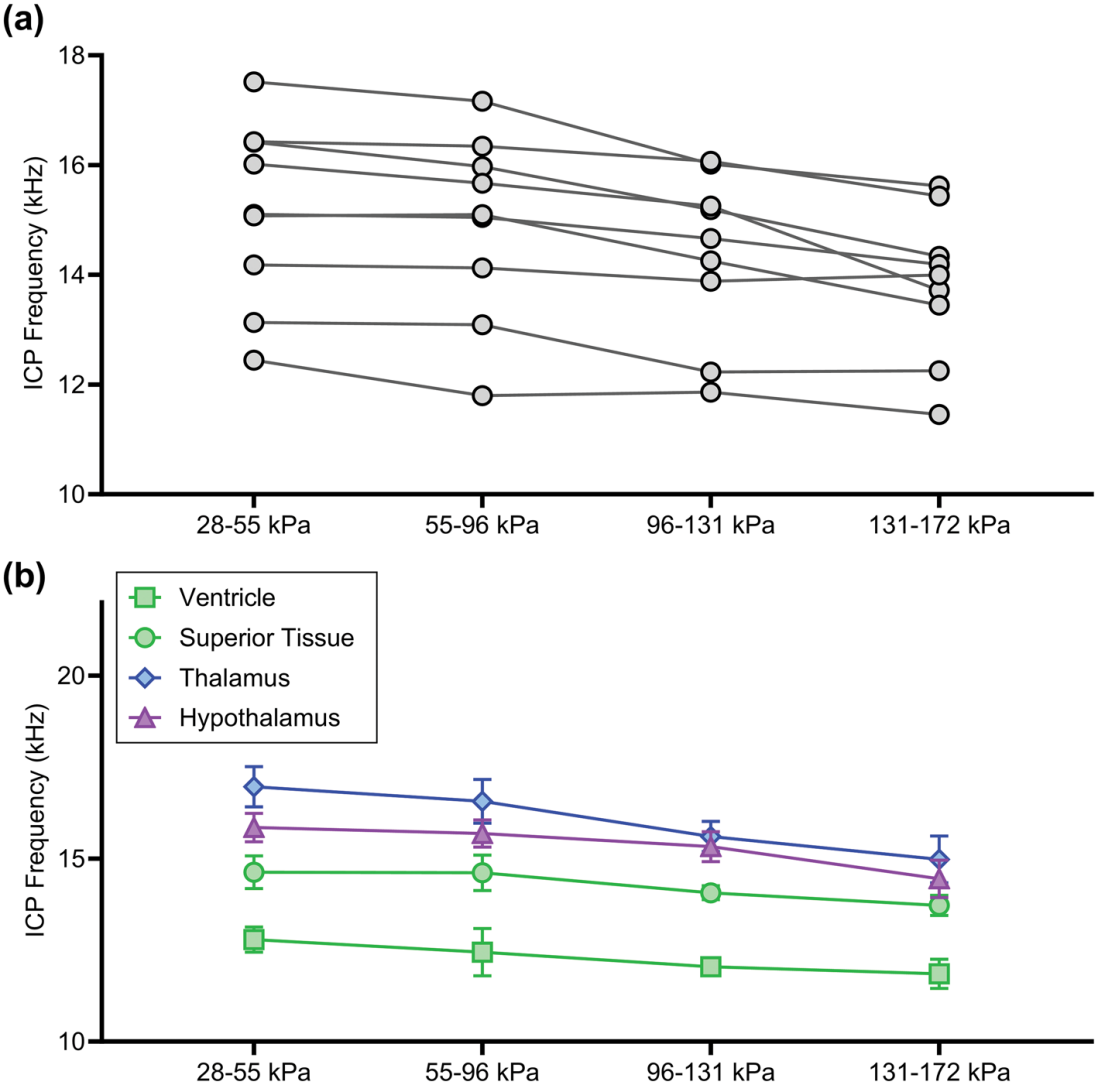
ICP frequency as a function of **a** SOP magnitude and **b** sensor location. ICP frequency significantly decreased with increasing SOP magnitude between 28 and 172 kPa (*p* < 0.0001). ICP frequency was significantly lower in the superior regions (green) where it was lowest in the ventricle compared to the thalamus and hypothalamus (*p* < 0.01)

The average frequency response directly following the peak ICP ranged from 11.5 to 17.5 kHz and was found to significantly decrease with increasing SOP magnitude between 28 and 172 kPa (*p* < 0.0001) (Fig. 8a). ICP frequency was also significantly influenced by sensor location (*p* < 0.01) (Fig. 8b). Sensors located in the superior brain regions showed a significantly lower ICP frequency compared to the midbrain thalamus and inferior hypothalamus regions. Further, the ventricles in the superior region had a significantly lower ICP frequency compared to the tissue in the superior region. Potential contributing factors include region-specific frequency modes of the cranium, variable cell densities, or disparities in tissue material properties.

## Discussion

*ICP profiles were measured using techniques capable of resolving regional disparities to best estimate brain mechanics in rats under head-on blast loading*. A sensor was inserted approximately 10 mm through the back of the skull (occiput) where it was then tightly secured using a resin to best model a rigid cantilever attachment. Similar boundary conditions were applied in Dal Cengio Leonardi et al. [15] where the occiput was selected, as opposed to the top of the skull, to minimize flexing at the insertion point and maintain as close to a static boundary condition as possible. We also wanted to preserve the flexural response over the top of the skull during blast since this was proposed as a mechanism of injury in rat models. Sensor placement through the occiput is likely more critical in rodent models compared to pig or human surrogate models because the surgical region covers a much larger ratio of the skull surface area.

Proper sealing around the sensor was also a critical step in maintaining a closed environment inside of the skull. During preliminary testing, bone cement was used to bond to a reference screw placed in the occiput, however, the bone cement was susceptible to cracking and fluid was found to seep between the occiput and the bone cement, breaking the seal (data not used). In the current study, the sensor insertion site was carefully inspected per animal following all blasting procedures to search for indicators of pressure leakage, known to influence pressure profiles in previous studies [12, 13, 16]. Therefore, the advantages of using the acrylate resin were that it was viscous, it bonded to the skull in the presence of fluid, and it eliminated the need for surgical implantation of the extra screw for stabilization. In addition to improved boundary conditions, this study utilized the SPR-524 sensor, which had a small size and reinforced coating that eliminated the need for cannula insertion. With these advantages, the surgical procedures were less invasive, it decreased the area of tissue damage, the resin more easily adhered to the sensor coating compared to a cannula, and it allowed for greater maneuverability when varying the sensor depth and angle of insertion, all of which were critical to achieve the spatial resolution shown in this work.

These techniques are not without challenges. Drilling through the occiput may increase risk of mortality due to the proximity of critical vasculature, which could lead to cerebral hemorrhage if disrupted. If these vessels are ruptured, the added blood volume within the brain may influence the change in ICP during blast. Additionally, stereotaxic procedures for accurate surgical placement of the sensors are only available for the top of the skull [43]. In this study, a less precise method of varying the depth and angle of insertion was applied and during preliminary testing it was noted that any errors in varying the angle led to large variations in sensor placement. Development of more precise surgical techniques through the occiput would reduce variability and improve confidence of sensor placement to allow for enhanced investigation of regional influences on the biomechanics during blast.

*The factor of ICP increase relative to the SOP varied linearly from superior to inferior region*. The data in this study showed that peak ICP was found to range from 1.30 to 2.14 times the SOP in a rat model depending on the relative brain location. A few studies previously reported little to no increase in peak ICP following blast [13, 20], which may be attributed to a pressure leak surrounding implantation of the pressure gauge [12, 16]. As all of the ICP profiles in this study resulted in a peak ICP/SOP greater than 1, we were confident that a solid pressure seal around gauge insertion was achieved. This was further confirmed through careful inspection of the sensor insertion site following all blasting procedures for each animal. The linear increase in peak ICP relative to the SOP was estimated in Elster et al. based on analysis of previously published experimental studies where peak ICP was approximated to be 1.3 times the SOP [44], which falls within the lower bound predicted in this study. It is important to consider that the physical presence of the sensor may influence the reported experimental measurements, particularly the magnitude of the ICP. This may explain some disparities in underestimation of peak ICP in computational models, which do not account for sensor effects. Additionally, if the experimental data were analyzed based on pressure sensors implanted in the top of the skull, compared to the occiput in this study, this would likely result in a lower ICP due to a dampened flexural response during blast. Further, the analysis by Elster et al. did not account for region-specific influences in the peak ICP.

Rubio et al. used a rat bTBI finite element model to predict regional differences in peak ICP [9]. Peak ICP was 1.17 times the SOP in the corpus callosum, 1.25 times the SOP in the hippocampus, and 1.27 times the SOP in the brainstem [9]. While these predictions accounted for brain region, the estimated peak ICP within these regions was underestimated. Estimations were calculated in part from previously developed finite element models [25, 26]. Directional changes in rat ICP profiles under frontal blast loading were modeled in Unnikrishnan et al. where the peak ICP was compared along the mid-coronal plane between the top, center, and bottom regions. Peak brain pressures at the center of the brain were predicted to be lower than in the top and bottom regions with less than 10% variation between all regions [26]. This is inconsistent with the experimental findings in this study where the peak ICP was lowest in the bottom and variation between regional peak ICP was greater than 30%. Continued use of these models to predict responses under different blast loading conditions, such as in Unnikrishnan et al. [25], could lead to confounded errors in future iterations, and is likely contributing to the underestimation of ICP in Rubio et al. [9]. Therefore, validation of finite element models solely through comparison of experimental and simulated peak ICP may not be sufficient and comparison of additional ICP waveform parameters should be considered.

Peak ICP was found to be driven mostly by the linear elastic skull/brain response between 28 and 172 kPa, which was least affected at low pressures and in the inferior brain regions in a head-on blast. In particular, peak ICP was lower in the hypothalamus compared to all other regions. We are the first to show this trend experimentally in rats where we hypothesize that the greater ICP in the superior brain regions is correlated with greater skull flexure at the top of the skull. The top of the rat skull is thinner than the sides or bottom, leading to greater mechanical input under blast loading and greater subsequent energy transfer, which is one of the main proposed mechanisms of bTBI [16]. Note that there was an exception in the trend from superior to inferior region (Table 2). The peak ICP the ventricle of subject 2 was 1.66 times the SOP, which was less than subjects in the thalamus (1.8-1.9 times the SOP). Upon further analysis, peak ICP was compared for subjects at similar vertical depths. The ICP was 2.14 times the SOP in the anterior ventricle (subject 3) compared to 1.66 times the SOP in the posterior ventricle (subject 2). Further, the ICP was 1.98 times the SOP in the anterior caudate putamen (subject 4) compared to 1.86 times the SOP in the posterior superior colliculus (subject 1). This may indicate a directional influence with greater peak ICP in the anterior regions compared to posterior regions, which was not compared statistically due to the low number of subjects in this study. This trend was shown in Unnikrishnan et al. where the peak ICP in the forebrain was 9% greater than in the midbrain and 14% greater than the hindbrain region [26]. Through the methods presented in this study, experimental analysis of spatial ICP profiles in small animal models could improve validation of these computational models for bTBI.

*Characterization of ICP profiles revealed that each phase varied independently as a function of both overpressure and brain region*. Previous studies quantified rat ICP characteristics such as the peak pressure [12, 14–21, 27], rise time [20], peak dP/dt [12, 27], frequency response [18], positive phase duration [17, 21], and positive impulse [15–18, 21], however, none of these studies have analyzed all profile characteristics. The data in this study showed that each metric varied independently as a function of SOP and brain region such that the ICP profiles uniquely developed based on the energy input, material, and geometric properties, also known as pressure fields. In particular, rise time, peak dP/dt, and frequency response were found to be most sensitive to regional differences and peak pressure, peak dP/dt, positive duration, positive impulse, and frequency response were sensitive to SOP magnitude. Therefore, reporting of a single measure (e.g., peak pressure) does not adequately represent the injury mechanics as a whole. We propose that common data elements for future studies characterizing ICP should incorporate measures of the peak pressure, rise time, peak dP/dt, positive duration, positive impulse, and frequency response.

*Analysis of the characterized ICP profiles provides evidence supporting the skull flexure injury mechanism*. Computational and experimental support for the direct cranial transmission theory was previously proposed as a major bTBI mechanism where the shock front transmits through the skull in normal compression/rarefaction waves that directly propagate throughout the brain tissue [1, 45]. Some may see this as intuitive because the ICP trace directly follows the surrounding shock wave profile. While the SOP visually correlated with the measured ICP characteristic curves when overlaid (Fig. 2a), studies have shown that this correlation is due to the pliability of the rat skull whereas the ICP characteristic curves in larger animal models, having stiffer skulls, deviate from the SOP wave characteristics [46, 47]. This deviation may be explained through analysis of phenomenological models where fluid-filled elastic shells generate time and spatially variant pressure fields within the internal fluid when exposed to a shock wave and the similar wave shape to the SOP is due to the elastic nature of the shell [47, 48]. This is consistent with skull flexure theory which is based on the idea that the air shock wave is almost entirely reflected from the skull but the resultant loading causes dynamic flexural deformation of the skull, which when closely coupled with surrounding tissue, imparts local forces that can be measured as time and spatially variant pressure fields within the brain [12, 45]. Parameterization of ICP spatial characteristics in this study demonstrates trends, particularly in the midbrain region, that are inconsistent with direct wave transmission theory and we propose that they are driven more by the skull/brain dynamics and geometry.

Under frontal blast loading, the ICP peak dP/dt and ICP frequency response were greatest in the thalamus region. According to the direct transmission theory, these measures would be expected to decrease in magnitude within the midbrain region compared to the frontal and superior regions. Given that this was not the case, we took a closer look at what could be influencing the distinct response in the thalamus region. Further inspection revealed a plateau feature in the ICP (see arrow in Fig. 3b) which was more distinct in the thalamus and increased in prominence with increasing blast magnitude. A similar plateau feature was visible in the ICP response of a fluid-filled spherical shell exposed to a blast wave where the plateau was most distinct in the middle of the sphere compared to the front and rear sections and was attributed to a flexural mode of the sphere during blast [48]. This plateau was also visible in Bolander et al. [12, 46] where ICP was measured under frontal blast loading in a rat model and correlated to the measured skull strain during blast. Although the ICP and associated skull strain were only analyzed at the onset of the shock front and the plateau was not discussed, data showed that the plateau similarly became more prominent with increasing blast magnitude and an anomaly was present in the skull strain beyond the onset of the shock front, which was also dependent on SOP magnitude. Therefore, we expect that this strain directly correlates to the plateau feature discussed here where we hypothesize that there is a secondary flexure mode contributing to regional pressure fields.

We must then consider how the presence of the plateau feature, increased dP/dt, and an increased frequency response make the thalamus and other regions in the center of the brain susceptible to unique injury mechanics. Based on evidence from phenomenological models, it is suspected that the skull flexure modes increase in complexity around the middle of the brain due to the semi-spherical geometry as well as influence from the jaw and orbital structures, as opposed to the smooth round surface along the top of the skull. This response is also potentially confounded with influence from varying material properties in the tissue or cell density between/within regions. Future studies are required to confirm the presence of a secondary flexure mode and better understand the region-specific implications on the injury response.

*Skull flexure is a dominant mechanism in rat models where the ICP frequencies measured in the brain are region-dependent for SOP magnitudes between 28 and 172 kPa*. Previous studies have provided visual evidence of this initial frequency (see Fig. 2a) where it was attributed to the flexural response of the skull under blast loading [12, 15, 16, 18, 27]. However, this was the first study to experimentally show that the measured frequency was sensitive to brain region. In particular, the thalamus and hypothalamus ICP frequencies ranged from 14 to 18 kHz, which was signifcantly higher than 13–15 kHz in the caudate putamen and superior colliculus. Further, the ICP frequency in the ventricles was found to be significantly lower than in the tissue, ranging between 11 and 13 kHz. Future work is required to uncover which variables are driving the disparities between brain regions (i.e., fluid/tissue material properties, cell density, region shape/geometry). Additionally, translational outcomes may be interpreted through reinforcement of the skull, particularly around the medial suture, where it would be anticipated to result in a significantly different ICP frequency response.

There also remains a need to investigate how this ICP frequency response may influence bTBI pathophysiology in rats. Skotak et al. measured a strong correlation between the ICP amplitude, oscillation frequency, and mortality in rats exposed to blast overpressures between 130 and 290 kPa where frequencies greater than 10 kHz corresponded with a higher mortality rate. It is important to note that it is possible that the ICP frequency increases above SOP magnitudes of 172 kPa where a second characteristic frequency closer to 20 kHz was detected for SOP between 250 and 290 kPa [18]. Further investigation may reveal how these mechanics may trigger a negative physiological response. In vitro models may have advantages where the cell response to changes in oscillation frequencies can be studied for a controlled amplitude. A recent study by Silvosa et al. tested the in vitro response of cerebral organoids and found that their neuronal network dynamics were altered when exposed to 300 Hz compared to 5 kHz [49]. Therefore, further modular testing of cellular response to frequencies ranging from 10 to 20 kHz may reveal how these frequencies contribute to the pathophysiology.

Region-specific ICP frequencies, independent of blast magnitude, may also provide insight on region-specific changes in blood–brain barrier (BBB) permeability. Regional changes in BBB permeability, particularly at low-level blasts, have long been a focus of understanding this injury mechanism. Specifically, alterations in the microvasculature pathology at low-level blasts is thought to have a selective vulnerability due to its otherwise normal brain parenchyma [50–52]. Nevertheless, BBB permeability was elevated in the hippocampus and thalamus at both low-level (72 kPa) and high-level (110 kPa) blast exposures [51]. Further, this permeability was greater than in the cortex. We hypothesize that higher ICP frequencies in the thalamus may correlate with increased BBB dysfunction where axial and radial diffusivity in the thalamus have been shown to be significantly increased following blast compared to other regions and the thalamus, striatum, and hippocampus experienced the greatest BBB disruption and BBB permeability [53, 54]. Based on the findings in this report: (1) Could the frequency be driving the pathophysiology more than the magnitude of the oscillations, particularly at low-level blasts? and (2) Is there a specific magnitude where the driving influence on the pathophysiology may shift from the frequency response to oscillation magnitude? Future research should investigate how the frequency response could be influencing vasculature permeability and may aid in understanding how low-level blast responses contribute to long-term pathology where the thalamus and hippocampus regions are notably more vulnerable.

It is not as far-fetched to think of how such frequencies may lead to damage when we examine another example of this in nature. Snapping shrimp generate low-magnitude, high frequency pulses under water in order to stun, injure, or even kill small prey including worms, goby fish, shrimp, or even small crabs. These animals, also known as pistol shrimp, contain a powerful claw that snaps shut at a high rate and propels a cavitation bubble towards its prey at approximately 1 μPa up to a distance of 1 m at frequencies between 10 and 200 kHz [55]. Kingston et al. recently found the orbital hood of snapping shrimp protects their brain against these frequencies and subsequently prevents short-term behavioral effects from this underwater shock wave exposure [56]. These findings demonstrate how high frequency shock wave exposure can result in neurotrauma in small animals at low pressure magnitudes. Future work should consider how increasing the magnitude of the shock wave while maintaining the high frequency input scales up in larger animals, especially given that these peak intracranial pressures are orders of magnitudes greater than for the pistol shrimp.

In conclusion, this study found a clear regional ICP variance in the rat brain during blast exposure. Given that these regional disparities were evident in such a small specimen suggests that dramatic, likely greater, differences may also be expected in the human. Identifying the exact characteristics of the stress waveform and in which areas is critical to interpreting injury outcomes, yet many bTBI publications still refer to ICP as if it was uniform; that idea goes back to the standard medical interpretation of ICP, i.e., a non-dynamic event. Evidence of regional variance, as shown in this report, is a key tenet of skull flexure theory. However, statistical analysis of regional influences was limited by the small number of animals per region examined. Therefore, further analysis is required to statistically analyze regional ICP disparities in larger populations, assess what may be driving these regional changes in ICP waveform (i.e., cell density, white/grey matter composition, brain region, skull/brain geometry, etc.), and subsequently determine how the regional changes in the ICP waveform (peak pressure, rise time, peak dP/dt, and frequency response) may be linked to changes in bTBI pathophysiology. A number of potential in vitro and in vivo approaches were discussed to build upon this knowledge and improve understanding of these mechanics. Additionally, it is imperative that future studies expand reporting of ICP characteristics to better define these unique spatial profiles and improve validation of finite element models.

## Supplementary Information

Below is the link to the electronic supplementary material.

Supplementary file1 (DOCX 54 kb)
