# Supplementary figures and images for: Spatial Intracranial Pressure Fields Driven by Blast Overpressure in Rats
Source: Ann Biomed Eng. 2024 Jun 8;52(10):2641–54. doi: 10.1007/s10439-024-03544-7 (PMC11402848; doi:10.1007/s10439-024-03544-7)

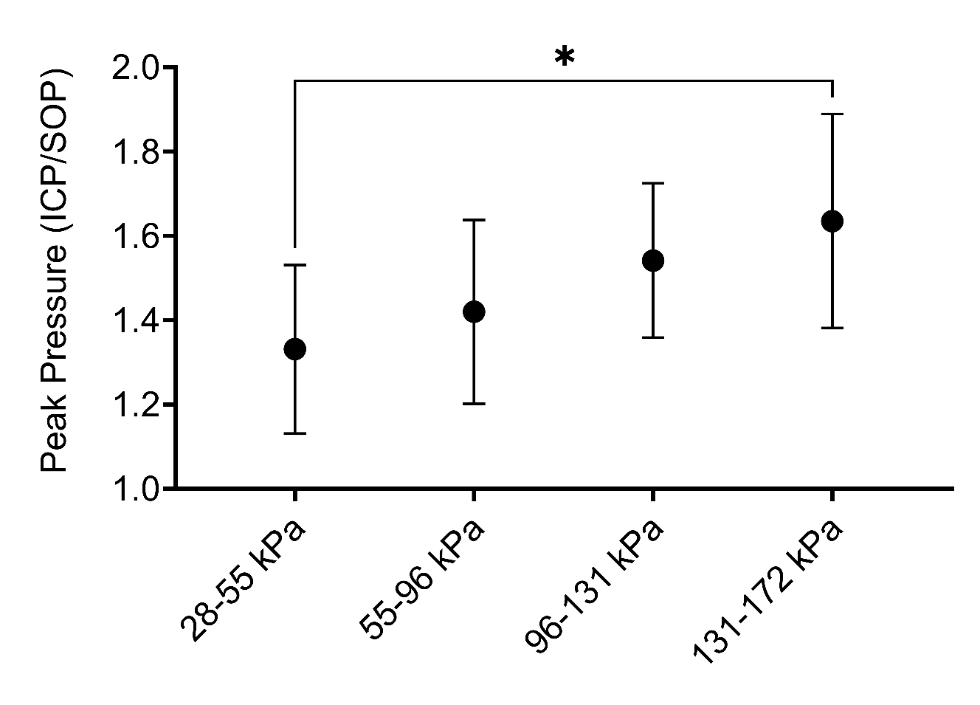


Supplementary Figure 1. Increasing ICP/SOP ratio with increasing SOP magnitude (*p<0.05).

Supplement: Supplementary file 1 — Supplementary file1 (DOCX 54 kb) [file 10439_2024_3544_MOESM1_ESM.docx]
